# Tislelizumab in combination with gemcitabine plus cisplatin chemotherapy as first-line adjuvant treatment for locally advanced or metastatic bladder cancer: a retrospective study

**DOI:** 10.1186/s12894-022-01083-8

**Published:** 2022-08-20

**Authors:** Xiang Ren, Yiqun Tian, Zhixian Wang, Jing Wang, Xing Li, Yisheng Yin, Ruibao Chen, Ying Zhan, Xiaoyong Zeng

**Affiliations:** Department of Urology, Tongji Hospital, Tongji Medical College, Huazhong University of Science and Technology, Wuhan, 430030 China

**Keywords:** Tislelizumab, Gemcitabine and cisplatin, Locally advanced or metastatic bladder cancer, Efficacy, Safety

## Abstract

**Background:**

Combining immune checkpoint inhibitors with chemotherapy can synergistically improve antitumor activity and are generally well tolerated. Recently, the efficacy and safety of combination therapy has been demonstrated for many cancers, including urothelial carcinomas. The aim of this retrospective pilot study was to evaluate the efficacy and safety of tislelizumab plus chemotherapy as first-line adjuvant treatment for locally advanced or metastatic bladder cancer.

**Methods:**

We conducted a retrospective analysis of 31 patients with locally advanced or metastatic bladder cancer from December 2020 to January 2022 with an Eastern Cooperative Oncology Group performance status of 0/1. Of the 31 patients, 14 patients received tislelizumab (200 mg i.v. every 3 weeks, Q3W) plus 21 days cycles of chemotherapy (gemcitabine, 1000 mg/m^2^ i.v. on days 1 and 8 of each cycle + cisplatin, 70 mg/m^2^ i.v. on day 2 of each cycle) (TGC) treatment and 17 patients received gemcitabine plus cisplatin chemotherapy (GC) treatment. All patients treated with bladder cytoreductive surgery and were treated for four 21 days cycles until disease progression or intolerable treatment-related adverse events (TRAEs). The objective progression-free survival (PFS), overall survival (OS), overall response rate (ORR), disease control rate (DCR), clinical benefit rate (CBR) and TRAEs were recorded and reviewed.

**Results:**

As of the cut-off date (March 25, 2022), PFS, OS, ORR, DCR, CBR and TRAEs were evaluated in 14 patients receiving combination therapy and 17 patients in the chemotherapy alone group. The median PFS was 36.0 [95% confidence interval (CI) 33.1–38.9] weeks in the TGC group and 29.0 (95% CI 25.4–32.6) weeks in the GC group [hazard ratio (HR) 0.15 (95% CI 0.04–0.55)]. In the GC group, the median OS was 48.0 (95% CI 39.7–56.3) weeks; the median OS was not yet mature for the TGC group [HR 0.26 (95% CI 0.07–0.94)]. Treatment with TGC resulted in improved DCR (TGC 71.4%; GC 65.0%) and CBR (TGC 64.3%; GC 52.9%) compared with GC. However, although higher incidences of grade ≥ 3 TRAEs were observed with TGC compared with GC (35.7% vs 23.5%), the difference was not statistically significant (*p* = 0.47).

**Conclusion:**

This study suggested that TGC provided survivors of locally advanced or metastatic bladder cancer with encouraging antitumor activity and was generally well tolerated.

## Background

Bladder cancer is among the most prevalent cancers worldwide, with around 430,000 new diagnoses each year [1]. Approximately 25% of newly diagnosed bladder cancer patients have muscle-invasive bladder cancer (MIBC) or metastatic disease [2, 3]. GC have been as a standard option for patients with locally-advanced or metastatic bladder cancer since a phase III trial comparing GC with methotrexate, vinblastine, doxorubicin, and cisplatin was conducted with a better safety profile [4]. Although bladder cancer is a chemosensitive disease and most patients with advanced or metastatic bladder cancer have disease control with first-line platinum-based chemotherapy, progression occurs in a short time due to chemotherapy resistance [4, 5]. Thus, there is an urgent need for other regimes that provide better survival outcome.

During the last few years, ICIs targeting programmed death-ligand 1 (PD-L1) and programmed death 1 (PD-1) have deeply changed the oncology field and become another pillar of cancer treatment. Following that, regimens that combine platinum-based chemotherapy and ICIs are appealing numerous studies for several reasons, such as platinum-based chemotherapy can induce immunomodulatory effects, thereby enhancing concomitant PD-L1 and PD-1 blockade [6, 7]. Now, a number of studies have demonstrated that the combination therapy could improve antitumor activity for many cancer types including non-small-cell lung cancer [8], esophageal squamous cell carcinoma [9], breast cancer [10], gastric cancer [11], and so on. Furthermore, in the field of urothelial carcinoma (UC), there are also various trials underway exploring or having initially obtained results that different kinds of ICIs in combination with platinum-based chemotherapy increase the clinical benefit, and these ICIs include durvalumab [12], nivolumab [13], pembrolizumab [14], atezolizumab [15]. Tislelizumab is another PD-1 monoclonal antibody drug that has also been shown to produce meaningful clinical benefits in patients with urothelial carcinoma and have a manageable safety profile [16].

Tislelizumab, a humanized IgG4 monoclonal antibody with high affinity and binding specificity for PD-1, can minimize binding to FcγRs on macrophages and reduce antibody-dependent phagocytosis which are a potential mechanism of T-cell clearance and resistance to anti-PD-1 therapy [17, 18]. In addition, compared with pembrolizumab and nivolumab, it shows higher affinity to PD-1 with an 100-fold slower off-rate than pembrolizumab and 50-fold slower off-rate than nivolumab [19]. Recently, several phase II trials evaluated the efficacy of tislelizumab combined with platinum-based chemotherapy with a result of that the combination could increase encouraging antitumor activity with manageable tolerability in patients with advanced lung cancer and esophageal squamous cell carcinoma or gastric/gastroesophageal junction adenocarcinoma [9, 20]. In the present study, we investigated the efficacy and safety of tislelizumab plus GC chemotherapy as first-line adjuvant therapy for locally advanced or metastatic bladder cancer.

## Methods

Patients who were diagnosed with locally advanced or metastatic bladder cancer at our center from December 2020 to January 2022 were reviewed in our retrospective study. The patients’ clinical and laboratory data were retrospectively retrieved by telephone and hospital medical case records. PD-L1 expression was assessed by immunohistochemistry with the VENTANA PD-L1 (SP263) assay at a central laboratory. Patients were considered PD-L1 + if immune cells (ICs) involved > 1% of the tumor area and ≥ 25% of tumor cells (TCs) or ICs had PD-L1 expression; or if ICs involved ≤ 1% of the tumor area and ≥ 25% of TCs or 100% of ICs expressed PD-L1 [21]. The selection criteria were as follows: a histologically or cytologically confirmed locally advanced or metastatic bladder cancer, an ECOG performance status of 0/1, and had undergone bladder cytoreductive surgery. Overall, 31 patients were recruited for the study; they were split into a TGC group comprising 14 cases and a GC group comprising 17 cases. Patients in the TGC group received tislelizumab (200 mg i.v. on days 1 each cycle) plus gemcitabine (200 mg/m^2^ i.v. on days 1 and 8 of each cycle) and cisplatin (70 mg/m^2^ i.v. on days 2 of each cycle), a 3 weeks cycle, for 4 cycles. Patients in the GC group received same schedule of gemcitabine and cisplatin.

All procedures followed the Good Clinical Practice guidelines and the principles of the Declaration of Helsinki. The study protocol was approved by the Ethics Committee of Tongji Hospital of Tongji Medical College, Huazhong University of Science and Technology.

Clinical efficacy and TRAEs were assessed by reviewing the patients’ medical histories and laboratory records. Tumor assessments were done at baseline and every 8 weeks thereafter (every 12 weeks after 24 weeks of treatment) until disease progression, unacceptable toxicity, or death, whichever occurred first. Clinical efficacy outcomes included PFS, OS, ORR, DCR and CBR in accordance with the Response Evaluation Criteria in Solid Tumors version 1.1. The TRAEs incidence and grade were assessed according to Common Terminology Criteria for Adverse Events Version 4.03 of the National Cancer Institute.

### Statistical analysis

Data were collected from patient enrolment (between December 2020 and January 2022) through to the final follow-up date (25 March 2022). Demographics/baseline disease characteristics and TRAEs were summarized using descriptive statistics. Between-group comparisons were conducted using chi-square tests. The Kaplan–Meier method was used to estimate the median survival follow-up, PFS and OS for each treatment group, and the 95% CI for the median PFS and OS was constructed using the Brookmeyer–Crowley methodology. PFS and OS were compared between trial groups using the stratified log-rank test. HRs were estimated using a stratified Cox regression model. For ORR, DCR, CBR and 95% CIs were constructed using exact method. All statistical analyses were performed using SPSS (version 22.0 IBM, Armonk, NY, USA) and R v.4.1.0 (www.r-project.org). Differences were considered statistically significant at *p* < 0.05.

## Results

### Patient demographics, baseline characteristics, and disposition

**Fig. 1:**
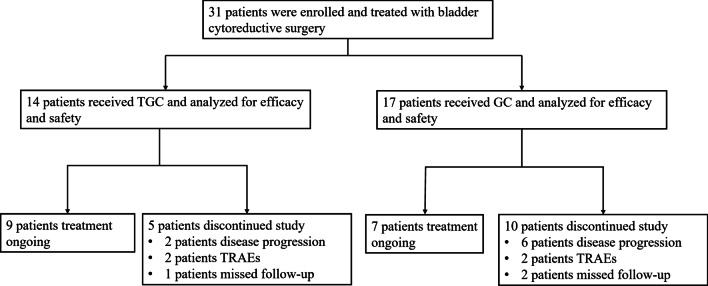
Patient disposition. TGC, tislelizumab plus gemcitabine and cisplatin; GC, gemcitabine and cisplatin; TRAEs, treatment-related adverse events

Between December 2020 and January 2022, a retrospective analysis of 31 patients with locally advanced or metastatic bladder cancer were enrolled in the study, of which 14 patients received TGC therapy and 17 received the GC therapy. As of March 25, 2022, 16 patients remained on treatment (Fig. 1). The reasons for discontinuation (n = 15) were progressive disease (TGC, n = 2; GC, n = 6), TRAEs (TGC, n = 2; GC, n = 2), and missed follow-up (TGC, n = 1; GC, n = 2). Baseline characteristics between the two groups were shown Table 1. There were no differences between the two groups with regard to the median age and gender. In addition, there were no differences in tobacco use, ECOG status, disease status and site of metastatic disease, and PD-L1 expression status between the two groups.

**Table 1:** Patient demographics and baseline characteristics

	TGC (n = 14)	GC (n = 17)	*p* value
*Age, years*			
< 65	6 (42.9)	7 (41.2)	0.93
≥ 65	8 (57.1)	10 (58.8)	
*Gender*			
Male	10 (71.4)	12 (70.6)	0.96
Female	4 (28.6)	5 (29.4)	
*Tobacco use*			
Never	5 (35.7)	7 (41.2)	0.77
Current or former	9 (64.3)	10 (58.8)	
*ECOG status*			
0	7 (50)	9 (52.9)	0.88
1	7 (50)	8 (47.1)	
*Disease status*			
Locally advanced	4 (28.6)	6 (35.3)	0.70
Metastatic	10 (71.4)	11 (64.7)	
*Site of metastatic disease*			
Lymph nodes only	2 (14.3)	2 (11.8)	0.86
Visceral^a^ ± lymph nodes	3 (21.4)	3 (17.6)	
Multiple metastatic sites	5 (35.7)	6 (35.3)	
*PD-L1 expression*			
TC < 50% and IC < 50%	6 (42.9)	8 (47.1)	0.73
TC ≥ 50% or IC ≥ 50%	2 (14.3)	3 (17.6)	
Unknown	6 (42.9)	6 (35.3)	

*TGC* tislelizumab plus gemcitabine and cisplatin, *GC* gemcitabine and cisplatin, *CI* confidence interval, *ECOG* Eastern Cooperative Oncology Group performance score, *PD-L1* programmed death ligand 1, *TC* tumor cell, *IC* immune cell

^a^Defined as one among of lung, liver, and bone metastases

### Antitumor activity of TGC

**Fig. 2:**
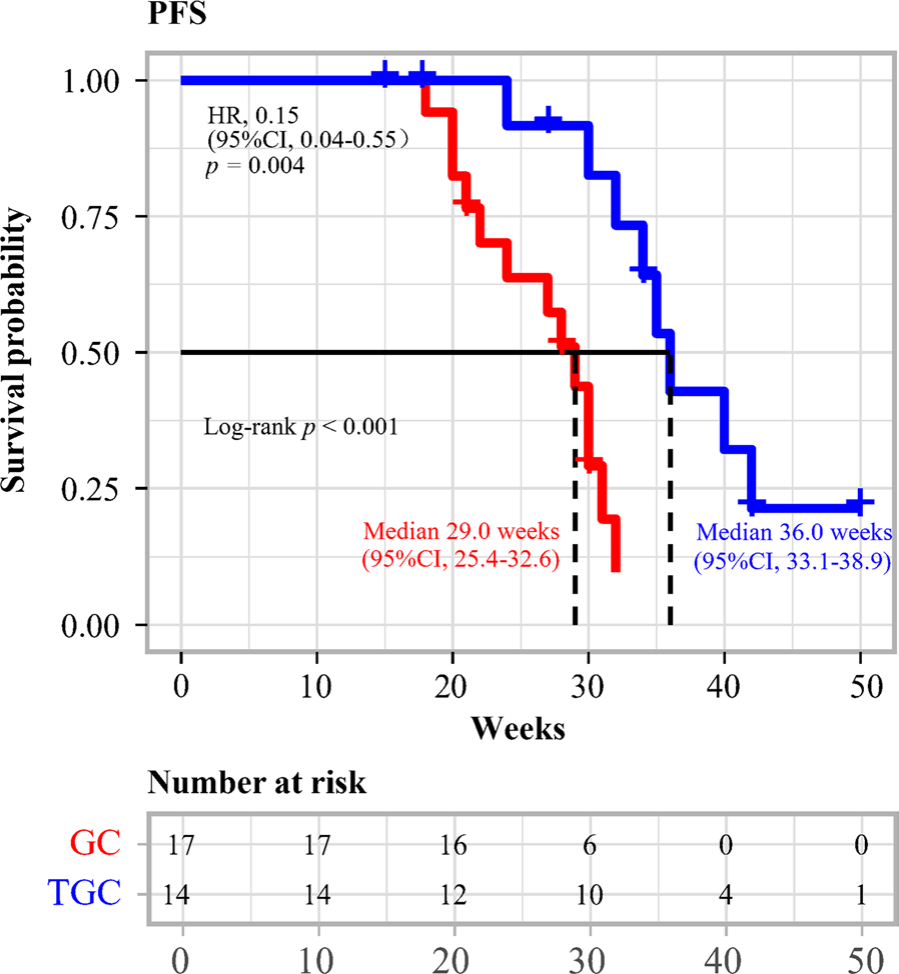
Kaplan–Meier estimates of the progression-free survival (PFS) in the tislelizumab in combination with gemcitabine plus cisplatin (TGC) and gemcitabine plus cisplatin (GC) groups. PFS, progression-free survival; TGC, tislelizumab plus gemcitabine and cisplatin; GC, gemcitabine and cisplatin; HR, hazard ratio; CI, confidence interval

**Fig. 3:**
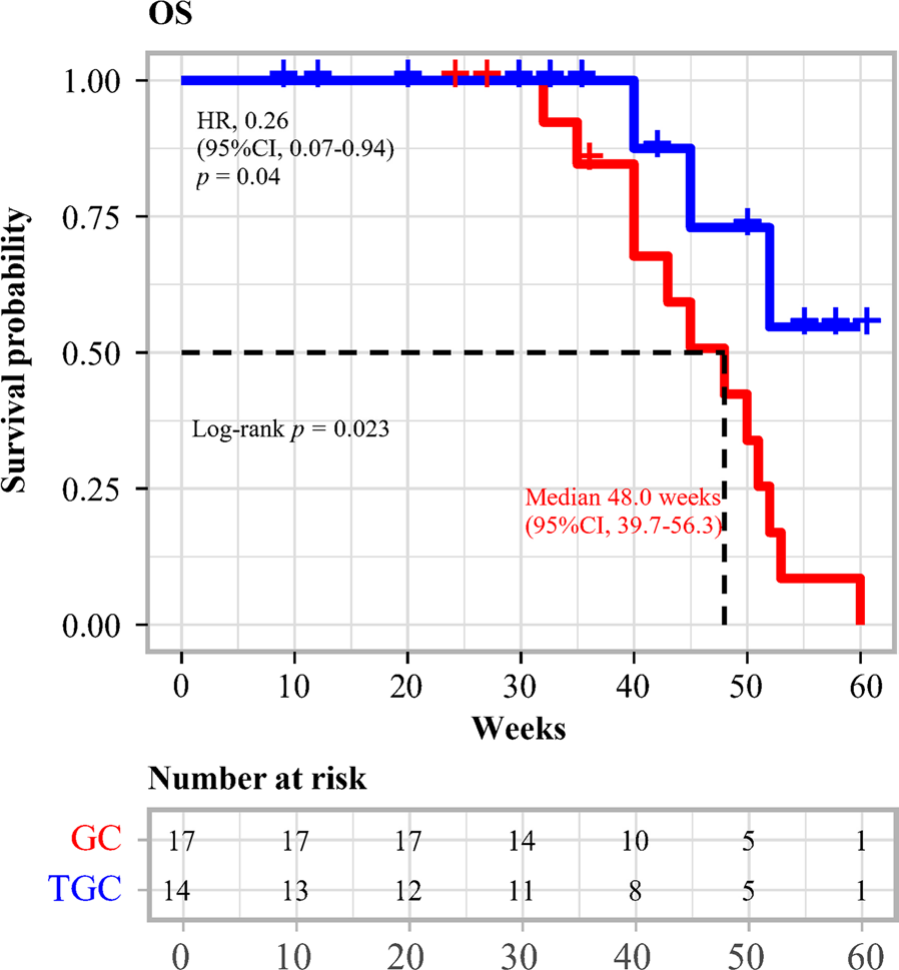
Kaplan–Meier estimates of the overall survival (OS) in the tislelizumab in combination with gemcitabine plus cisplatin (TGC) and gemcitabine plus cisplatin (GC) groups. OS, overall survival; TGC, tislelizumab plus gemcitabine and cisplatin; GC, gemcitabine and cisplatin; HR, hazard ratio; CI, confidence interval

As of March 25, 2022, all 31 patients were included in the efficacy evaluable set. The median survival follow-up was 54.3 (range, 9.0–63.1) weeks in the TGC group and 52.1 (range 20.0–61.2) weeks in the GC group. The median PFS in the TGC group was 36.0 (95% CI 33.1–38.9) weeks, which was longer than that of the GC group 29.0 (95% CI 25.4–32.6) weeks [HR 0.15 (95% CI 0.04–0.55); *p* = 0.004] (Fig. 2). Furthermore, the median OS in the GC group was 48.0 (95% CI 39.7–56.3) weeks. Despite the long median survival follow-up, the median OS in the TGC group had not been reached [HR, 0.26 (95% CI 0.07–0.94); *p* = 0.04] (Fig. 3). In addition, the PFS rate at 24 weeks was 91.7% (95% CI 77.3–100.0%) in the TGC group and 63.7% (95% CI 44.2–91.8%) in the GC group; and the OS rate at 48 weeks was 72.9% (95% CI 46.8–100.0%) in the TGC group and 42.3% (95% CI 21.8–82.0%) in the GC group.

Of all patients, the confirmed objective responses in the TGC group was higher than in the GC group (TGC 57.1%; GC 47.1%), though the difference was not statistically significant (ORR, *p* = 0.59). Moreover, more patients in the TGC group, compared with the GC group, had a complete response (TGC 14.3%; GC 11.8%) and a partial response (TGC 42.9%; GC 35.3%). In addition, treatment with TGC resulted in improved disease control rate (TGC 71.4%; GC 64.7%) and clinical benefit rate (TGC 64.3%; GC 52.9%) compared with GC. However, no statistically significant difference was observed between patients with different therapy regimes (DCR, *p* = 0.96; CBR, *p* = 0.54) (Table 2).

**Table 2:** Disease response per RECIST v1.1

	TGC (n = 14)	GC (n = 17)	*p* value
Best overall response, n (%)			
Objective response rate, % (95% CI)	57.1 (27, 87)	47.1 (21, 74)	0.59
Complete response	2 (14.3)	2 (11.8)	
Partial response	6 (42.9)	6 (35.3)	
Stable disease	5 (35.7)	5 (29.4)	
Progressive disease	1 (7.1)	2 (11.8)	
Missing or unevaluable	0 (0)	2 (11.8)	
Disease control rate, % (95% CI)	71.4 (44, 98)	64.7 (39, 90)	0.96
Clinical benefit rate, % (95% CI)	64.3 (36, 93)	52.9 (26, 79)	0.54

*RECIST v1.1* Response evaluation criteria in solid tumors version 1.1, *TGC* tislelizumab plus gemcitabine and cisplatin, *GC* gemcitabine and cisplatin, *CI* confidence interval

### Safety and tolerability

All 31 patients (100%) experienced at least one TRAE (Table 3). The most common TRAEs in the TGC group were anemia (57.1%), decreased appetite (57.1%), nausea/vomiting (50.0%), and thyroid disorders (42.9%); and the most common TRAEs in the GC group were anemia (47.1%), nausea/vomiting (35.3%), decreased appetite (29.4%), increased blood creatinine (29.4%) and decreased WBC count (29.4%). However, although higher incidences of grade ≥ 3 TRAEs were observed in the TGC group, the difference was not statistically significant (TGC, 35.7%; GC, 23.5%, *p* = 0.47). The most frequently reported grade ≥ 3 TRAEs in the TGC group were decreased appetite (28.6%), nausea/vomiting (28.6%), anemia (21.4%) and thyroid disorders (21.4%); and the most frequently reported grade ≥ 3 TRAEs in the GC group were nausea/vomiting (17.6%) and increased blood creatinine (17.6%) (Table 3). No deaths related to TRAEs were observed.

**Table 3:** Treatment-related adverse events occurring in ≥ 10% of patients with locally advanced or metastatic bladder cancer in either group

	TGC (n = 14)		GC (n = 17)	
	Any grade	Grade ≥ 3	Any grade	Grade ≥ 3
Patients with ≥ 1 AEs, n (%)	14 (100)	5 (35.7)	17 (100)	4 (23.5)
Anemia	8 (57.1)	3 (21.4)	8 (47.1)	2 (11.8)
Pyrexia	5 (35.7)	1 (7.1)	4 (23.5)	2 (11.8)
Decreased WBC count	5 (35.7)	2 (14.3)	5 (29.4)	2 (11.8)
Decreased neutrophil count	4 (28.6)	2 (14.3)	3 (17.6)	1 (5.9)
Decreased platelet count	4 (28.6)	2 (14.3)	3 (17.6)	1 (5.9)
Increased ALT	2 (14.3)	0	2 (11.8)	0
Increased AST	2 (14.3)	0	2 (11.8)	0
Fatigue	4 (28.6)	1 (7.1)	4 (23.5)	1 (5.9)
Decreased appetite	8 (57.1)	4 (28.6)	5 (29.4)	2 (11.8)
Rash	2 (14.3)	0	2 (11.8)	0
Nausea/vomiting	7 (50.0)	4 (28.6)	6 (35.3)	3 (17.6)
Thyroid disorders	6 (42.9)	3 (21.4)	0	0
Increased blood creatinine	4 (28.6)	2 (14.3)	5 (29.4)	3 (17.6)

*TGC* tislelizumab plus gemcitabine and cisplatin, *GC* gemcitabine and cisplatin, *AEs* adverse events, *WBC* white blood cell, *ALT* alanine aminotransferase, *AST* aspartate aminotransferase

## Discussion

This retrospective study investigated the antitumor activity and safety of tislelizumab in combination with GC chemotherapy for patients with locally advanced or metastatic bladder cancer compared with GC chemotherapy alone. As of the data cut-off date, this study showed that patients treated with tislelizumab plus GC had longer progression-free survival and overall survival, and a higher proportion of patients achieving an overall response and disease control than did those treated with GC. In addition, no significant differences were observed regarding safety and tolerability between patients with tislelizumab plus GC chemotherapy and patients with GC chemotherapy alone, though higher incidences of grade ≥ 3 TRAEs were observed in the TGC group.

Gemcitabine and cisplatin combination chemotherapy regime is the typical treatment of choice for patients with locally advanced or metastatic bladder cancer. The median PFS and OS in first-line chemotherapy typically observed can range from approximately 6.8–8.8 months and 11.0–15.5 months; the ORR approximately 41–43.6% [22–24]. In this study, the GC group patients had confirmed the results of the previous analysis despite only 17 cases [22–24].The TGC group provided better efficacy with regard to PFS, OS, ORR and DCR compared to GC group. This could be, as discussed above, platinum-based chemotherapy can induce immunomodulatory effects, which enhanced PD-L1 and PD-1 blockade [6, 7], or alternatively it could be due to absence of clinical cross-resistance between therapeutic classes and a number of patients received treatment beyond first-line therapy [25, 26]. In addition, six patients in the TGC group were not evaluated for PD-L1 expression due to economic conditions or other reasons, which might lead to changes in the final results. Because a recent phase 2 trial showed that tislelizumab produced clinical benefits in patients with PD-L1-positive urothelial carcinomas [16]. However, some trial results suggested that PD-L1 status is not useful when adding chemotherapy [14]. So the study demonstrated that the combination therapy could produce a meaningful antitumor activity and there is a need to further validate the specific mechanism in following studies.

IMvigor130, a phase 3 study of atezolizumab combined with platinum-based chemotherapy in patients with metastatic UC resulted in PFS and OS were 8.2 months and 16.0 months, respectively [15]. Within the TGC group of this study, the median PFS was 36.0 weeks, and despite a median survival follow-up of approximately 54.3 weeks, median OS was yet immature at the time of data cutoff. The prolongation of PFS or the possible prolongation of OS might be partially due to the fact that patients in the TGC group of the study received bladder cytoreductive surgery, while the patients in IMvigor130 trial did not. Furthermore, all patients in the TGC group received cisplatin as platinum-based chemotherapy, whereas a partial of patients received carboplatin-based chemotherapy in IMvigor130 trial, where cisplatin could have a better antitumor response than carboplatin [27]. On the other hand, as tislelizumab shows higher affinity to PD-1 than pembrolizumab and nivolumab, whether tislelizumab binds better to PD-1 than atezolizumab binds better to PD-L1 potentially [19], or there are some other reasons that are still unclear. Taken together, the results of tislelizumab plus GC chemotherapy supported encouraging antitumor activity in patients with advanced locally advanced or metastatic bladder cancer.

The TRAEs profile, there were no new safety signals observed with TGC combination therapy, which was consistent with those of previous tislelizumab in combination with chemotherapy trials in other advanced solid tumors [9, 20]. Most of TRAEs in the TGC group of the study were associated with chemotherapy, except for thyroid disorders. A recent PURE-01 trial demonstrated that thyroid dysfunction was the most common all-grade immunotherapy-related adverse events (18%) [28]. Just as some patients may experience retarded immune-related adverse events, including hypothyroidism, adrenal insufficiency and increased liver enzymes [29], satisfactorily, there were no adrenal insufficiency appearing and only two patients with elevated liver enzymes. And the relationship between elevated liver enzymes and tislelizumab was uncertain, because two patients in the GC group had also the same degree of elevated liver enzymes. The majority of TRAEs considered related to tislelizumab by the investigator were generally mild-to-moderate severity. Importantly, although higher incidences of grade ≥ 3 TRAEs were observed in the TGC group, the group did not result in a higher number of patients discontinuing, or a higher number of deaths than in the GC group. The results showed that tislelizumab in combination with chemotherapy was generally tolerated and manageable in the any grade TRAEs.

To our knowledge, this was the first retrospective report of GC chemotherapy as a single agent or in combination with tislelizumab in patients with locally advanced or metastatic bladder cancer as the first-line therapy. Since this was a retrospective study and collected patient information was not always complete, the population size and duration of follow-up were limited, which led to large confidence intervals and the antitumor activity or safety could not be determined well. However, the results of tislelizumab alone in the treatment of urothelial carcinoma and its combination with platinum-based chemotherapy in the treatment of other tumors could confirm and support that our research results provided valuable real-life information about the treatment and the prognosis of these patients. So a prospective, larger, multicenter, more comprehensive-demographics, open-label, phase III study should be conducted in great detail to assess efficacy and safety of tislelizumab in combination with platinum-based chemotherapy in patients with locally advanced or metastatic urothelial carcinoma.

## Conclusion

This retrospective study suggested that GC chemotherapy plus tislelizumab can provide survivors of locally advanced or metastatic bladder cancer with encouraging antitumor activity and are generally well tolerated. However, this was a single institute experience with a limited number of patients and a limited time. The new clinical trials should offer further guidelines for clinical treatment.

## Data Availability

All data supporting the study are presented in the manuscript or available upon request. Further inquiries can be directed to the corresponding author (Prof. Zeng, miwai@163.com).

## Abbreviations

ICIs: Immune checkpoint inhibitors
ECOG: Eastern cooperative oncology group
TGC: Tislelizumab in combination with gemcitabine plus cisplatin
GC: Gemcitabine plus cisplatin chemotherapy
TRAEs: Treatment-related adverse events
PFS: Progression-free survival
OS: Overall survival
ORR: Overall response rate
DCR: Disease control rate
CBR: Clinical benefit rate
HR: Hazard ratio
MIBC: Muscle-invasive bladder cancer
PD-L1: Programmed death-ligand 1
PD-1: Programmed death 1
UC: Urothelial carcinoma
ICs: Immune cells
TCs: Tumor cells
